# Changes in sex ratio at birth among immigrant groups in Sweden

**DOI:** 10.1186/s41118-018-0036-8

**Published:** 2018-09-03

**Authors:** Eleonora Mussino, Vitor Miranda, Li Ma

**Affiliations:** 1 Demography Unit—Department of Sociology, SUDA—Stockholm University, SE-106 91 Stockholm, Sweden; 2 Statistics Sweden, Stockholm, Sweden; 3 Karlstad University, Karlstad, Sweden

**Keywords:** Sex ratio at birth, Sex preference for children, Sex selection of children, Immigrants, Sweden

## Abstract

What happens when citizens from societies with strong son preference culture migrate to countries in which preference for having a child of each sex prevails? Using data from Swedish population registers, we investigate the sex ratio at birth by parity and the sex composition of previous children in Sweden. Our results showed that women with Chinese, Korean, and Indian background had a substantially elevated sex ratio at the third parity if previous children were both girls. Strikingly, this skewed ratio became less pronounced after 2000, suggesting a shift for a more neutral sex preference for children among these groups in the new century.

## Background

The sex ratio at birth (SRB) is defined as the number of male births per female births. Without external interventions, among newborns, there is a slight excess of boys over girls and the natural SRB lies at approximately 1.05. In some Asian societies, however, the SRB since the mid-1980s has been strongly skewed toward boys (Gu and Roy 1995; Guilmoto 2009). For instance, the SRB was estimated at 1.16 in China in 2014 and at 1.10 in India in 2012 (Guilmoto 2015). Such skewed patterns are also evident in some Eastern European and Caucasian countries (Bongaarts 2013; Duthé et al. 2012; UNFPA 2012).

A series of factors has been reported to impact the ratio of boys to girls among newborns. For instance, this ratio has been linked to demographic factors such as maternal age and birth order (e.g., James 1996; Jacobsen et al. 1999); biological factors such as mother’s hormonal levels around the time of the conception (James 1996, 2004; Van Larebeke et al. 2008); environmental conditions such as maternal or paternal exposures to pesticide or pollution in general (Van Larebeke et al. 2008; Terrell et al. 2011); and parents’ socioeconomic status such as religion, occupation, income, and education (Teitelbaum and Mantel 1971; Markle 1974; Murata and Imaizumi 1982; Chahnazarian 1988; Almond et al. 2013). Recent studies have linked the large imbalances in the SRB to the consequences of sex-selective abortion among certain populations (Seth 2010). This practice emerged when strong son-preference norms were combined with extensive access to ultrasonography, a technology that can determine the sex of the fetus (Bongaarts 2013). Since the 1980s, the decline in the desired number of children per couple and the availability of prenatal diagnosis technology have reinforced sex selection of children, and consequently a skewed SRB (Chen et al. 2013). The World Health Organization (2011, p. 12) defines the unbalanced sex ratios at birth as “an unacceptable manifestation of gender discrimination against girls and women and a violation of their human rights.” Nonetheless, the prevalence of son preference may change across time and contexts. For example, Korea’s economic development and social efforts in promoting gender equality smoothed the previously skewed SRB in Korean society (Chung and Das Gupta 2007, Ma 2016).

Interestingly, skewed SRBs are also observed among certain immigrant groups in countries where the SRB of the native population ranges around the natural value. For example, a skewed SRB toward boys is documented among Indian-born mothers in England (Dubuc and Coleman 2007); among Chinese, Korean, and Indian parents in the USA (Almond and Edlund 2008); and among South and East Asian immigrants in Canada (Almond et al. 2013). These findings suggest that preference for sons over daughters may prevail among certain populations, even after they have migrated to a context with different welfare systems and gender norms.

Sweden as a universal welfare state is an interesting case to study. On the one hand, the “liberal” and “individualistic” context allows for individual choices. On the other hand, the Swedish social environment promotes social norms that are conducive to gender equality and higher fertility. Mason (1997) argues that a society’s gender system arguably influences parents’ sex preference for children. In societies with a high level of gender equality, one should expect a lower preference for the sex of offspring. In Sweden, most parents find two children the ideal family size and the preferred sex-composition is one son and one daughter (Andersson et al. 2006). Among couples with two children, those with only boys are more likely to continue childbearing than those with two girls, signaling an emergence of girl preference in Swedish society (Andersson et al. 2006, 2007).

What happens when citizens from societies with strong son preference culture migrate to countries in which preference for having a child of each sex prevails or even emerging daughter preferences are common? Do they adjust their childbearing preference behavior, or do they maintain the cultural values of their home country? Previous studies have documented the persistence of son preference among Finish immigrants in Sweden (Andersson et al. 2007). We have little knowledge about whether immigrants from other areas with son-preference culture, such as East and South Asia, maintain their son-preference childbearing behavior in Sweden. This study addresses these issues by exploring the differences of sex ratios at birth between different immigrant groups and the native Swedes by birth order and the sex composition of previous children over time. We expect that findings of this study will enrich existing knowledge on immigrants’ childbearing behavior in destination countries with evidence from Sweden. Further, we expect that this study will contribute to literature on gender discrimination and give implications for future policy development regarding gender equality.

## An overview of sex preferences for children

There is a vast amount of literature on sex preferences for children in less developed countries (Bongaarts 2013; Sen 1990). A group of studies has inferred patterns of sex preference in a society by comparing parity progression rates according to the sex composition of their offspring (Arnold 1997; Rahman and DaVanzo 1993). A significantly higher birth rate among couples with only daughters than among those with only sons would be a sign of preference for sons, for instance. The underlying interpretation is that, in societies that value sons more than daughters, couples would try harder to have at least one son in the family than at least one daughter. Another group of studies has focused on the analysis of SRB. An SRB that is highly skewed toward boys would be an indication of son preference and a possible sex-selective action as prenatal sex discernment, sex selection abortion, or sex-selective pre-implantation.

Preference for sons has been observed in East and South Asian societies, such as China, South Korea (or Korea), and India, resulting in imbalances in SRB, as well as much higher parity progression ratios in families with daughters and no sons (Arnold 1997). A commonality of these societies is their kinship system: the persistence of strong patrilineal and patrilocal family systems, where sons have a much greater life cycle economic utility for their parents than daughters. The main productive assets of families would be passed on to sons, who would most often reside in or near their parents’ house and take care of them after marriage (Das Gupta et al. 2003; Murphy et al. 2011; Ma 2016). Parents often invest less in girls’ education and their upbringing (Gao 2003). A woman’s primary duty is to bear sons for her husband’s lineage (Chung and Das Gupta 2007). Thus, the incentive to have a son drives couples to continue childbearing if the previous children are daughters (Ma 2016; Poston 2002).

Discrimination against girls has shifted from the postnatal discrimination mode to the prenatal discrimination mode with the availability of sex-selective technology since the mid-1980s, which sharply increases SRB in East and South Asian areas (Das Gupta et al. 2003). In addition, the decline of fertility at the national level may boost the rise of SRB. For example, the fertility decline in China, largely due to the country’s population restriction policy implemented in 1979 (later known as the one-child policy), plays a positive role in the rise of SRB; couples may make the best use of the modern technology to ensure that their first or second birth is a boy (Chu 2001). Goodkind (2011) finds that the SRB of first-order births in China approached the normal SRB levels in 2005, whereas that of second births exceed 1.50. Similarly, the consequence of fertility decline in the rise of SRB is also observed in India in the 1980s and the 1990s (Das Gupta and Bhat 1997).

Nonetheless, recent studies record a decline in the preference for sons in urban China and South Korea. In China, relative to the extremely high levels of SRB in rural areas, the levels of SRB in metropolitan areas are much lower (Guilmoto 2009). Fong (2002) reports an increase of girls’ empowerment in urban areas of China where the one-child policy was strictly implemented. The author argues that singleton daughters in urban areas enjoy unprecedented parental support as they meet no competition from brothers. In South Korea, the SRB increased from a normal range to 1.15 from 1980 to 1990. It shifted to a plateau during the mid-1990s, followed by a steady decline. As of 2015, with the SRB (1.07) returning almost to the biologically normal range, Korea completed its SRB transition (Chung and Das Gupta 2007; Guilmoto 2009; Lutz et al. 2018). The reduction of SRB can be reflected in individual’s continued childbearing behavior. In the 1980s, women who bore a girl for the first birth had substantially higher likelihood of having a second child than did women who had a boy. However, this gap has reduced over time. Around the 2000s, the difference disappeared (Ma 2016). Economic development, social policies in reducing gender inequality, and normative change arguably contribute to the decline of son preference (Chung and Das Gupta 2007; Ma 2016). In comparison, in some other Asian societies with higher level of fertility and son preferences, such as Sri Lanka, no skewed SRB has been observed (Guilmoto 2009).

In contrast to East and South Asian societies, it has been argued that in the contemporary European context, children are not seen mainly as a source of economic security, but valued largely for social and psychological reasons (Hoffman and Hoffman 1973). However, a recent study by Bongaarts (2013) finds evidence that son preference exists in the former Soviet Union countries (i.e., Armenia, Azerbaijan, Georgia, Kazakhstan, and Uzbekistan). In Moldova and Armenia, the sex of the child(ren) that have already been born plays a strong role in determining the transition to a second or third birth. Those with only girls have a substantially higher likelihood of wanting another child (Billingsley 2011). Evidence of sex ratio imbalance has also been found in Albania and in the Balkans in general, with a skewed SRB biased toward boys (UNFPA 2012).

Sex preferences in childbearing behavior and its implications for fertility decisions have also been a topic of research for developed societies in the West. There are studies on this issue for North America (Pollard and Morgan 2002), Denmark (Jacobsen et al. 1999), Germany (Hank and Kohler 2002), and Sweden (Andersson et al. 2006, 2007). These studies mainly look at parity progression contingent on sex composition of prior births and have provided strong evidence of parents’ preference to have at least one child of each sex: parents with only daughters or only sons are more likely than others to have another child. In other words, parents from these developed societies do not lack gender preference for children. Instead, they prefer to have at least one boy and one girl in the household. In addition, research based on large-scale register data of the Nordic countries revealed evidence of emerging daughter preferences in childbearing. In particular, couples that have two sons are more likely to have a third child (presumably trying for a daughter) than those couples that have two daughters. This pattern holds for Denmark, Norway, and Sweden, but not for Finland (Andersson et al. 2006, 2007).

When migrants come to host countries, they might bring along the cultural values of their home countries. Arnold (1997) insists that immigrants from countries with strong preference for sons over daughters would adhere to the same practices in the host countries. A few studies on sex preferences for the children of immigrant women with an Asian background indicate elevated SRB, especially for higher-order births (see Almond and Edlund 2008 and Abrevaya 2009 for the USA; Ray et al. 2012 and Almond et al. 2013 for Canada; Dubuc and Coleman 2007 for the UK; González 2014 for Spain; Singh et al. 2010 for Norway; Ambrosetti et al. 2015, Blangiardo and Rimoldi 2012, and Meldolesi 2011 for Italy; González 2014 for Spain; and Verropoulou and Tsimbos 2010 for Greece). These results are consistent with observations in their country of origin.

Nonetheless, the practice of son preference in reproductive behavior among certain immigrant groups may change across time. Tønnessen and colleagues (2013) demonstrate a changing trend of SRB among Indian immigrants in Norway. In the period between 2006 and 2012, Indian-born women gave birth to more girls than boys at higher birth orders. This result contradicts findings for earlier periods and other Western societies, implying that later immigrant cohorts or immigrants who have resided in the host country for a long time may have new sex preferences for children.

## Immigration to Sweden

Today, the share of foreign-born represents about 18% of the Swedish population (SCB 2018). However, throughout much of its history, Sweden was a country of emigration, mostly to North America. Since the early 1930s, with the return of the Swedes from North America, an era of immigration began. During World War II, the number of immigrants to Sweden began to increase. The Nordic and Baltic regions were the major resources of immigrants. After the war, Sweden started receiving immigrants from other European countries to overcome the strong shortage of labor in the labor market (Allwood et al. 2006; Brochmann and Hagelund 2012).

Labor immigration decreased in the 1970s and 1980s. Instead, refugee immigration and family reunion immigration increased. Social unrest in South America, Middle East, and Eastern Africa resulted in an increasing number of refugees coming to Sweden (Allwood et al. 2006). Subsequently, the percentage of immigrants from non-European countries increased from approximately 10% in the mid-1970s to 50% in the 1980s (Allwood et al. 2006).

During the 1990s and 2000s, one important feature of immigration to Sweden was family reunion. Forty-five percent of the immigrants who came for family reunification in 1986 were from Europe. The figure decreased to 16% in 2014. In contrast, the corresponding figure for immigrants with an Asian background increased from 26 to 54% (Migrationverket 2016). Asia has gradually become the largest source of immigrants in Sweden (Allwood et al. 2006).

It is noteworthy that, in the late 2000s and 2010s, the composition of Asian immigrants had a drastic change from being characterized by mainly adopted children to economic migrants. Students became an important group, accounting for more than 40% of immigrants coming from China (Statistics Sweden 2016a, 2016b). Likewise, migrant laborers and students have become a dominant group among immigrants from India (Statistics Sweden 2016a).

## Sweden: a multicultural society

Immigrants bring cultural traditions from their home country. These cultural traditions and values might be more likely to be maintained if the destination country is tolerant of cultural diversity. Sweden formulated a multicultural immigrant policy in the mid-1970s, encouraging cultural diversity and equality within the area of culture and education, which means that immigrants and their children have the right to “retain their own language, develop their own cultural activities, and maintain contact with their original country” (Borevi 2012, p. 41). In other words, equality and freedom of choice are promoted, and the immigrants can maintain their own distinctive cultural identity.

As mentioned, the sources of immigrants to Sweden have become more diversified over time. Arriving in tandem with immigrants is their cultural capital, including language, values, ways of thinking, and their preferences for the sex of their children. Given the strong son preference culture in East and South Asia, and based on the findings regarding the elevated SRB among immigrants from these areas in different hosting contexts, we make the following hypotheses for the context of Sweden.^1^

- Hypothesis 1: Immigrants from areas with a strong son preference culture, such as East and South Asia (including China, Korea, and India), should have higher SRB than other immigrant groups and the native-born Swedes, especially at higher order births if previous children are girls. The theory behind this hypothesis is the “cultural persistence” (Almond et al. 2013) of immigrants’ norms and values related to fertility and family behaviors.
- Hypothesis 2: The SRB of our target immigrant groups may change over time (we look at both period and duration effect). The theory behind this hypothesis is that the gender egalitarian value of Swedish society and its generous welfare system may lead to more neutral parental sex preferences for children.

## Data

The data come from the Swedish population register, which is kept by the Swedish national bureau of statistics. These administrative data started to be digitalized in the 1960s and include all individuals who were registered as residents in Sweden, as well as births, deaths, and migrations (Statistics Sweden 2003, 2011).

In this study, the sex ratio at birth, or SRB, is used as a measure for parents’ sex preference for children. We examine the SRB for both foreign-born and native mothers in Sweden by birth order and sex composition of previous children. A skewed SRB (above 1.06) is considered a result of sex-selection of children due to preference of having a son. Previous studies for other contexts found that son-biased sex ratios are more common at third or higher order births among couples that previously only had daughters (e.g., Almond and Edlund 2008). This distortion has been argued as indirect evidence for sex-selective abortions (Dubuc and Coleman 2007).

Our analysis focuses on the period between 1980 and 2015 and includes births of all women born in 1954 or later who ever resided in Sweden (native and foreign born). We started the analysis from 1980 because obstetric ultrasonography and the possibility to discern the sex of a fetus became more widely available from that time onward. Nonetheless, data preparation used information on births that occurred as early as the 1960s. This step was necessary in order to construct complete birth histories. Mothers who had multiple births at any point in their lifetime were not included in the study and only live births were considered.

Given that our main interest is to look into childbearing behavior after migration, only births that occurred in Sweden were used to compute our dependent variable—SRB. Births that occurred before migration were taken into account when constructing birth histories. This can help us better capture the association between the sex composition of previous child(ren) and the SRB.

Children born before migration are recorded in the population register if the children immigrated to Sweden at some point in their lives. Although some births that occurred before migration to Sweden might not be included in the population register (Andersson 2004), available evidence suggests that the number of such cases should be relatively small among women in their childbearing ages. The register data showed that, in the period from 1968 to 2015, approximately three quarters of all women who migrated to Sweden during their reproductive ages (e.g., before age 45) entered the country before age 30. This means that their children were young and likely to have accompanied them to Sweden.

Moreover, a nationally representative survey shows that migrating to Sweden during one’s childbearing ages and leaving a child behind in the mother’s home country is an extremely rare event. In 2009, less than 2% of all foreign-born women between the ages of 20 to 40 had a biological or adopted child who did not co-reside with them.^2^ Therefore, it can be expected that the percentage of biological children who actually stayed behind in the mothers’ home country is even lower. In sum, we have strong evidence that the administrative data provides a fairly accurate representation of the birth histories of both native- and foreign-born mothers.

Table 1 presents the number of births by birth order and country of birth of mothers in categories. Southeast Asia includes Brunei, Cambodia, East Timor, Indonesia, Laos, Malaysia, Myanmar, the Philippines, Singapore, Thailand, and Vietnam. The Horn of Africa region includes Djibouti, Eritrea, Ethiopia, and Somalia. The total studied population includes over 3.1 million births, of which approximately 2.5 million occurred to Swedish-born mothers and 0.6 million to foreign-born mothers. During the period under study, there were approximately 1.5 million first births, 1.2 million second births, and 0.4 million third births.

**Table 1:** Number of births, by birth order, and country/region of birth of the mother, in thousands: Sweden, 1980–2015

Country/region	1st births	2nd births	3rd births	Total
Sweden	1238.7	1002.5	346.6	2587.8
Iran, Iraq	30.8	25.3	11.6	67.7
Former Yugoslavia	27.3	24.4	10.5	62.1
Southeast Asia	16.1	12.4	4.7	33.2
Horn of Africa	13.2	11.2	7.8	32.3
Turkey	10.5	9.4	5.8	25.7
Poland	12.2	9.2	2.6	24.0
China, Korea, India	11.8	8.0	1.9	21.7
Syria	6.7	6.2	4.3	17.2
Afghanistan, Pakistan	4.6	3.5	1.9	10.0
Rest of Europe	73.0	59.5	22.3	154.8
Americas and Oceania	20.0	16.1	6.5	42.6
Rest of Asia	16.1	13.3	7.5	36.8
Rest of Africa	12.3	10.0	5.4	27.8
Total	1493.3	1211.0	439.4	3143.8

Source: Swedish population register

## Results

The standard biological range of the SRB lies between 1.04 and 1.06 male births per female birth (UNFPA 2012). We computed point estimates and confidence intervals to investigate whether the SRB is outside of this expected range among mothers born in different countries. Table 2 shows the SRB for first and second births by mothers’ country of birth. The SRB for second births are shown separately according to the sex of the first child.

**Table 2:** Sex ratio at birth, 1st and 2nd births, by sex of previous children, and country/region of birth of the mother: Sweden, 1980–2015

Country/region	1st birth		2nd birth, first was a boy		2nd birth, first was a girl	
	Sex ratio	95% C.I.	Sex ratio	95% C.I.	Sex ratio	95% C.I.
Sweden	1.06	(1.06–1.06)	1.06	(1.05–1.06)	1.06	(1.06–1.07)
Iran, Iraq	1.06	(1.03–1.08)	1.06	(1.03–1.10)	1.05	(1.01–1.08)
Former Yugoslavia	1.08	(1.06–1.11)	1.06	(1.03–1.10)	1.05	(1.01–1.09)
Southeast Asia	1.05	(1.02–1.09)	1.06	(1.01–1.12)	1.10	(1.04–1.15)
Horn of Africa	1.04	(1.00–1.07)	1.05	(1.00–1.11)	1.09	(1.04–1.15)
Turkey	1.07	(1.03–1.11)	1.06	(1.00–1.12)	1.06	(1.00–1.12)
Poland	1.06	(1.03–1.10)	1.04	(0.98–1.10)	1.11	(1.04–1.17)
China, Korea, India	1.03	(1.00–1.07)	1.05	(0.98–1.11)	1.06	(1.00–1.13)
Syria	1.08	(1.03–1.13)	1.09	(1.02–1.17)	1.09	(1.02–1.17)
Afghanistan, Pakistan	1.05	(0.99–1.11)	1.06	(0.97–1.17)	1.02	(0.93–1.13)
Rest of Europe	1.06	(1.04–1.08)	1.06	(1.03–1.08)	1.07	(1.04–1.09)
Americas and Oceania	1.05	(1.02–1.08)	1.04	(1.00–1.09)	1.06	(1.01–1.10)
Rest of Asia	1.04	(1.01–1.08)	1.07	(1.02–1.12)	1.04	(0.99–1.09)
Rest of Africa	1.09	(1.05–1.13)	1.07	(1.01–1.12)	1.09	(1.03–1.15)

95% confidence intervals shown in parentheses. Source: Swedish population register

Table 2 does not show remarkably skewed SRB at either the first or the second birth among any groups of mothers. Most point estimates are within the natural range between 1.04 and 1.06. Some values are slightly higher than expected, with the most extreme cases being the sex ratio for second births among mothers born in Poland who had a daughter in their first birth: 1.11 male births per female birth (95% CI [1.04, 1.17]). The point estimates also indicate male-heavy sex ratios for second births among mothers born in Africa, Southeast Asia, and Syria who had a daughter in their first birth. However, this observation cannot be taken as statistical evidence of sex preference or sex selection of children because the estimated confidence intervals contain the expected natural range.

Table 3 shows the sex ratio at third birth by sex composition of previous children and mothers’ country of birth. The SRB at third birth is particularly pronounced among mothers from China, Korea, and India. Among mothers born in these countries who had two daughters, there were 1.32 male births per female birth (95% CI [1.11, 1.57]).

**Table 3:** Sex ratio at birth, 3rd births, by sex of previous children, and country/region of birth of the mother: Sweden, 1980–2015

Country/region	Two boys		Mixed		Two girls	
	Sex ratio	95% C.I.	Sex ratio	95% C.I.	Sex ratio	95% C.I.
Sweden	1.06	(1.04–1.07)	1.06	(1.05–1.07)	1.06	(1.04–1.07)
Iran, Iraq	1.06	(0.99–1.14)	1.03	(0.98–1.09)	1.08	(1.00–1.16)
Former Yugoslavia	1.04	(0.96–1.12)	1.09	(1.03–1.16)	1.10	(1.02–1.18)
Southeast Asia	1.04	(0.93–1.15)	1.09	(1.01–1.19)	1.13	(1.01–1.27)
Horn of Africa	1.04	(0.95–1.13)	1.02	(0.96–1.08)	1.01	(0.92–1.11)
Turkey	1.12	(1.01–1.24)	0.99	(0.92–1.07)	1.07	(0.97–1.19)
Poland	0.97	(0.84–1.12)	1.00	(0.90–1.12)	1.06	(0.91–1.24)
China, Korea, India	0.97	(0.81–1.15)	1.13	(0.99–1.29)	*1*.*32*	(*1*.*11*–*1*.*57*)
Syria	0.94	(0.84–1.05)	1.02	(0.94–1.11)	1.08	(0.95–1.21)
Afghanistan, Pakistan	1.14	(0.96–1.36)	0.98	(0.86–1.11)	0.92	(0.77–1.10)
Rest of Europe	1.04	(0.99–1.09)	1.09	(1.05–1.13)	1.02	(0.97–1.07)
Americas and Oceania	1.06	(0.97–1.16)	1.10	(1.02–1.18)	1.06	(0.96–1.16)
Rest of Asia	1.04	(0.95–1.14)	0.99	(0.92–1.05)	1.08	(0.99–1.18)
Rest of Africa	1.03	(0.93–1.15)	0.99	(0.91–1.07)	1.00	(0.90–1.11)

95% confidence intervals shown in parentheses. Source: Swedish population register

Italicized data are the confidence interval exceeds that of the natural range

Among all of the groups that were studied, this is the only case in which the confidence interval exceeds that of the natural range. The SRB was within the natural range among mothers from those same countries who previously had two sons (0.97, 95% CI [0.81, 1.15]) or a son and a daughter (1.13, 95% CI [0.99, 1.29]). This general pattern provides support for the hypothesis that there is a cultural persistence of a preference for sons over daughters among mothers born in this region, even after they migrate to Sweden. In contrast, the SRBs of mothers who migrated to Sweden from other regions are closer to the natural range.

**Fig. 1:**
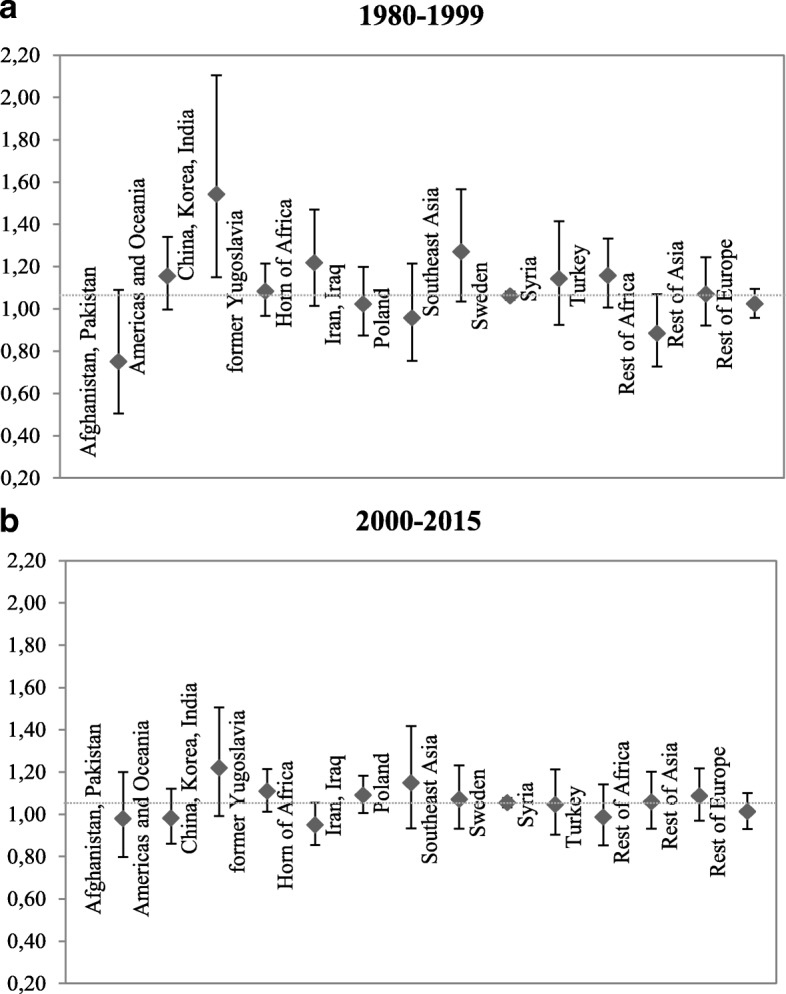
Sex ratio at birth, third births, mothers who had two daughters, by country/region of birth of the mother, over calendar time, Sweden. **a** 1980–1999. **b** 2000–2015. Notes: The dotted line indicates sex ratio at birth equal to 1.06 Source: Swedish registers

To test whether the SRB of our target immigrant groups changed over calendar time, we split the observation period into two intervals: 1980–1999 and 2000–2015. Figure 1a shows that during the period between 1980 and 1999, the SRB for women from China, Korea, and India is the most biased with almost 1.6 male births per female birth, while that for the other groups does not statistically differ from the natural value. Women from Southeast Asia seem to have a visibly skewed SRB as well, though the point estimate is not statistically significant. However, when we look at the SRBs of the period between 2000 and 2015 in Fig. 1b, the significance level of the computed results disappear for all groups. The SRB of our target group declined to 1.22. This finding deserves special attention. It implies that despite the increasing accessibility to sex selection methods in the new century, the immigrants from areas with a culture that strongly prefers sons (e.g., China, Korea, and India) somewhat shift for a more neutral sex preference for children.

This pattern might also suggest a selection effect. The composition change of our target immigrant groups may help interpret the pre- and post-2000 differences in SRB. Relative to the earlier cohorts of immigrants from China, Korea, and India, the later cohorts of immigrants from the same regions are better educated (Statistics Sweden 2016b) and, thus, may be more egalitarian (Kishor and Gupta 2009; Shu 2004) and more likely to practice similar childbearing behaviors as the native population do. Nonetheless, it might also be the result of the recent decline in the preference for sons in urban China and South Korea. However, the change of SRB over calendar time can be attributed to women who have resided for longer duration in the country (i.e., they probably arrived as children), as well as to the most recent flows. Due to small number of cases within each duration category, we did not further split the estimation for pre- and post-2000 separately. Nevertheless, Table 4 displays the changes of SRB based on duration of residence in Sweden for our target mother group for the period 1980–2015.

**Table 4:** Sex ratio at birth, 3rd births, for women coming from China, Korea, and India, by years in Sweden: Sweden, 1980–2015

Years in Sweden	Sex ratio	95% C.I.	Observations
Up to 5	1.18	(0.76–1.83)	87
6–10	1.71	(1.21–251)	130
10–20	1.93	(1.23–3.26)	79
More than 20	1.08	(0.83–1.40)	233

95% confidence intervals shown in parentheses. Source: Swedish registers

The table shows a reverse U-shape of sex ratio at third birth by the duration of residence for women with Chinese, Korean, and Indian backgrounds. In particular, the SRB of those who have resided in Sweden for more than 20 years, who had migrated probably as adopted child, approximates the natural value. For those women, growing up in Sweden (and probably in a Swedish family) promotes sex preference similar to the Swedish society. This pattern not only reflects an adaptation to the gender-neutral preferences for children in Sweden, but also confirms a selection of the most recent cohort effect.

### Limitation

The data administration restriction limits our ability to examine the SRB of mothers from China, Korea, and India separately, though we are aware that these three societies have various socioeconomic and cultural differences and are differently represented in Sweden.^3^ However, because of their long history of son-preference culture, previous studies (e.g., Almond and Edlund 2008) have already grouped the three countries together.

In addition, Sweden is currently a country with the largest population of international adoptees in Europe and the highest per capita rate of international adoptions in the world (Von Borczyskowski et al. 2006). Adoption from Asia gradually started from the 1970s. Having been exposed to the Swedish values since childhood, adoptees may be more likely to practice the neutral sex preference for children as the native Swedes do. In other words, our estimation for our target immigrant groups might be downward biased if the adoptees are included in analysis. Had we excluded the adoptees, our estimated SRB for our target group might have been higher. Future research needs to address this issue when more adoptees with Asian background complete their reproductive career.

## Discussion and conclusion

This paper contributes to the literature by studying the parental sex preferences for children of immigrants in Sweden. Our observation covers the period between 1980 and 2015. Following the existing literature, we assess the SRB of different immigrant groups and native population in Sweden by parity and sex composition of the previous child(ren). In particular, we look at the SRB of immigrant mothers from China, Korea, and India, exploring whether mothers from areas with a strong son preference culture maintain their home country’s son preference culture in a universal welfare state, where a neutral sex preference for children prevails.

Our results show that despite their emergent preference for having daughters (Andresson et al.2007), the Swedish-born women do not show any sign of sex selection of children, nor do the majority of the immigrant groups residing in the country, with the exception of women from China, Korea, and India. We find that the SRB for women with Chinese, Korean, and Indian backgrounds is substantially skewed at the third parity if previous children are both girls, confirming earlier findings for other destination countries, such as Dubuc and Coleman (2007) in the UK, Abrevaya (2009) and Almond and Edlund (2008) in the USA, and Almond et al. (2009) in Canada. The result confirms hypothesis 1 and it is consistent with the “cultural persistence” theory that women coming from countries with strong son-preference culture tend to maintain this culture in the destination countries.

However, the prevailing gender egalitarian values in Sweden seem to have a direct impact on the childbearing behavior of immigrants from China, Korea, and India, which confirms hypothesis 2. Relative to their statistically significant boy-skewed SRB for the period between 1980 and 1999, their SRB for the period between 2000 and 2015 is much lower. This period change suggests that immigrant mothers from son-preference culture with the exposure to Sweden’s gender egalitarian value and universal welfare have weakened their preference to have a son in the most recent years or at least they did not resort to sex selection of the child. Similar results have also been found in Norway (Tonnessen et al. 2013). Nevertheless, it might suggest that, relative to earlier cohorts of immigrants from son-preference culture, later cohorts of immigrants from the same area might be a more selected group with higher level of education and more gender egalitarian values.

This positive effect of residing in Sweden on reducing SRB in the most recent years is also evident when we compare the SRB for Chinese, Korean, and Indian mothers in Sweden with that for women in their home countries. Women from China, Korea, and India in Sweden exhibit, for the third parity, 1.32 boys for 1 girl when they have already had 2 girls during our observation period. The same figures for their home countries were 2.25, 1.36, and 1.39, respectively (Zeng et al. 1993; Park and Cho 1995; Jha et al. 2006).^4^ Looking at different destination countries (Table 5) with different sex preference norms and different welfare regimes, we find a consistent cultural persistence on son preferences among our target group. However, this persistence seems to be stronger for Chinese, Korean, and Indian mothers in Anglo-Saxon countries, such as the UK, USA, and Canada, and for the same group in familistic welfare states such as Greece, Italy, and Spain. In the Nordic countries (e.g., Sweden and Norway), which are characterized by universalistic and gender equalitarian welfare states and liberal abortion legislation,^5^ even if a still-skewed boy preference is present, the SRB are drastically lower. This result supports our conclusion on the importance of a gender egalitarian and generous welfare state to offset the skewed SRB.

**Table 5:** Current studies on sex ratio at birth, 3rd births with two girls for previous children of immigrant women from China, Korea, and India in different destinations

Destination country	Period	Origin of migrants	SRB	Authors, year	Data
Sweden	1980–2015	Chinese, Korean, and Indian	1.32	Mussino et al. 2018 (this study)	Registers
USA	2000	Chinese, Korean, and Indian	1.51	Almond and Edlund 2008	Census
Canada	2001–06	Indian	1.90	Almond et al. 2013	Census
Canada	2001–06	Chinese, Korean, and Vietnamese	1.39	Almond et al. 2013	Census
Norway	1969–2005	Indians	1.29	Singh et al. 2010	Retrospective cohort study
Italy	2008–09	Chinese	1.44	Ambrosetti et al. 2015	Survey data
Italy	2008–09	Indian	2.23	Ambrosetti et al. 2015	Survey data
Italy	2011	Chinese	1.87	Blangiardo and Rimoldi 2012	Survey data
Italy	2011	Indian	4.25	Blangiardo and Rimoldi 2012	Survey data

## Footnotes

1 We are aware that skewed sex ratio at birth and the assumption of sex selection is only one indicator of gender preference (see our overview of sex preferences for children). Sex preference might influence fertility in different ways. For example, parents may have a gender preference, but instead of resorting to abortion, they may have an additional birth (see Andersson et al. 2006; Andersson et al. 2007).

2 The point estimate is 1.6% with a 95% confidence interval of [0.05, 3.23]. A detailed description of the survey has been published by Statistics Sweden (2009).

3 In 2017, among women in reproductive ages from China, Korea, and India in Sweden, 44% were from China, 37% from India, and 18% from South Korea. In the period 2000–2017, women from China almost triplicated, from India duplicated and from Korea increased only 12%, respectively (www.scb.se).

4 The numbers for China, Korea, and India came from data for 1989–90, 1991, and 1997, respectively (see Zeng et al. 1993; Park and Cho 1995; and Jha et al. 2006).

5 In Sweden, abortion is legal up to the end of the 18th week of pregnancy. Any woman (registered or unregistered in the health system) can ask for and receive an abortion without having to give a reason. Between the 18th and 22nd weeks of pregnancy, abortion is permitted only if the National Board of Health and Welfare (Socialstyrelsen) gives special permission for a reason, such as if the woman’s health is at risk. After the 22nd week, it is not allowed in any circumstances.

## References

[CR1] Abrevaya J (2009). Are there missing girls in the United States? Evidence from birth data. American Economic Journal: Applied Economics.

[CR2] Allwood J, Edebäck C, Myhre R (2006). An analysis of immigration to Sweden. European intercultural workplace project—work package 3.

[CR3] Almond D, Edlund L (2008). Son-biased sex ratios in the 2000 United States census. Proceedings of the National Academy of Sciences.

[CR4] Almond D, Edlund L, Milligan K (2009). O Sister, Where Art Thou? The Role of Son Preference and Sex Choice: Evidence from Immigrants to Canada.

[CR5] Almond D, Edlund L, Milligan K (2013). Son preference and the persistence of culture: evidence from south and east Asian immigrants to Canada. Population and Development Review.

[CR6] Ambrosetti, E., Ortensi, L.E., Castagnaro, C. Attili, M. (2015). “Sex imbalances at birth in migratory context: evidence from Italy” GENUS, LXXI (No. 2-3), 29-51.

[CR7] Andersson G (2004). Childbearing after migration: fertility patterns of foreign-born women in Sweden. International Migration Review.

[CR8] Andersson G, Hank K, Ronsen M, Vikat A (2006). Gendering family composition: sex preferences for children and childbearing behavior in the Nordic countries. Demography.

[CR9] Andersson G, Hank K, Vikat A (2007). Understanding parental gender preferences in advanced societies: lessons from Sweden and Finland. Demographic Research.

[CR10] Arnold F (1997). Gender preferences for children. Demographic and Health Surveys Comparative Studies 23.

[CR11] Billingsley S (2011). Second and third births in Armenia and Moldova: an economic perspective of recent behavior and current preferences. European Journal of Population.

[CR12] Blangiardo, G.C., Rimoldi, S. 2012. 100 milioni di bambine mancano all’appello nel mondo. E in Italia?, Neodemos 2012. http://www.neodemos.info/articoli/100-milioni-di-bambine-mancano-allappello-nel-mondo-e-in-italia/. Accessed 24 Oct 2012.

[CR13] Bongaarts J (2013). The implementation of preferences for male offspring. Population and Development Review.

[CR14] Borevi K, Brochmann G, Hagelund A, Borevi K, Jonsson HV, Petersen K (2012). Sweden: the flagship of multiculturalism. Immigration policy and the Scandinavian Welfare State 1945–2010.

[CR15] Brochmann G, Hagelund A, Brochmann G, Hagelund A, Borevi K, Jonsson HV, Petersen K (2012). Welfare State, Nation and Immigration. Immigration policy and the Scandinavian welfare state 1945–2010.

[CR16] Chahnazarian A (1988). Determinants of the sex ratio at birth: review of recent literature. Social Biology.

[CR17] Chen Y, Li H, Meng L (2013). Prenatal sex selection and missing girls in China: evidence from the diffusion of diagnostic ultrasound. Journal of Human Resources.

[CR18] Chu J (2001). Prenatal sex determination and sex-selective abortion in rural Central China. Population and Development Review.

[CR19] Chung W, Das Gupta M (2007). The decline of son preference in South Korea: the roles of development and public policy. Population and Development Review.

[CR20] Das Gupta M, Bhat PNM (1997). Fertility decline and increased manifestation of sex bias in India. Population Studies.

[CR21] Das Gupta M, Zhenghua J, Bohua L, Zhenming X, Chung W, Hwa-Ok B (2003). Why is son preference so persistent in east and South Asia? A cross-country study of China, India and the Republic of Korea. The Journal of Development Studies.

[CR22] Dubuc S, Coleman D (2007). An increase in the sex ratio of births to India-born mothers in England and Wales: evidence for sex-selective abortion. Population and Development Review.

[CR23] Duthé G, Meslé F, Vallin J, Badurashvili I, Kuyumjyan K (2012). High sex ratios at birth in the Caucasus: modern technology to satisfy old desires. Population and Development Review..

[CR24] Fong V (2002). China’s one-child policy and empowerment of urban daughters. American Anthropologist.

[CR25] Gao X (2003). Women existing for men: Confucianism and social justice against women in China. Race, Gender and Class.

[CR26] González L (2014). Missing girls in Spain.

[CR27] Goodkind D (2011). Child undererporting, fertility, and sex ratio imbalance in China. Demography.

[CR28] Gu, B., & Roy, K. (1995). Sex ratio at birth in China, with reference to other areas in East Asia: what we know. *Asia-Pacific Population Journal, 10*(3 (1995)), 17–42. 12290692

[CR29] Guilmoto CZ (2009). The sex ratio transition in Asia. Population and Development Review.

[CR30] Guilmoto CZ (2015). The masculinization of births. Overview and current knowledge. Population.

[CR31] Hank, K., & Kohler, H. –. P. (2002). Gender preferences for children revisited: new evidence from Germany. *MPIDR Working Paper*, 2002–2017.

[CR32] Hoffman, L.W. and Hoffman, M.L. (1973). The Value of Children to Parents. In: Fawcett, J.T. (ed.). Psychological perspectives on population. New York: Basic Books: 19−76.

[CR33] Jacobsen R, Moller H, Mouritsen A (1999). Natural variation in the human sex ratio. Human Reproduction.

[CR34] James WH (1996). Further concepts on regulators of the sex ratio in human offspring: Interpregnancy intervals, high maternal age and seasonal effects on the human sex ratio. Human Reproduction.

[CR35] James WH (2004). Further evidence that mammalian sex ratios at birth are partially controlled by parental hormone levels around the time of conception. Human Reproduction.

[CR36] Jha P, Kumar R, Vasa P, Dhingra N, Thiruchelvam D, Moineddin R (2006). Low male-to-female sex ratio of children born in India: National Survey of 1.1 million households. Lancet.

[CR37] Kishor, S., & Gupta, K. (2009). *Gender equality and Women’s empowerment in India*, *National Family Health Survey (NFHS-3) India, 2005–06* (p. 138). Ministry of Health and Family Welfare Government of India.

[CR38] Lutz W, Goujon A, Samir KC, Stonawski M, Stilianakis N (2018). Demographic and human capital scenarios for the 21st century2018 assessment for 201 countries.

[CR39] Ma L (2016). Female labor force participation and second birth rates in South Korea. Journal of Population Research.

[CR40] Markle GE (1974). Sex ratio at birth: values, variance, and some determinants. Demography.

[CR41] Mason KO, Jones GW, Douglas RM, Caldwell JC, D’Souza RM (1997). Gender and Demographic Change: What Do We Know?. The continuing demographic transition.

[CR42] Meldolesi, A. (2011). “Mai nate. Perché il mondo ha perso 100 milioni di donne”, Milano: Mondadori Università.

[CR43] Migrationverket. (2016). *Residence permits granted 1986-2014—family reunification*. Migrationverket. https://www.migrationsverket.se

[CR44] Murata M, Imaizumi Y (1982). An analysis of the sex ratio and occupational class in Japan. Journal of Biosocial Science.

[CR45] Murphy R, Tao R, Lu X (2011). Son preference in rural China: patrilineal families and socioeconomic change. Population and Development Review.

[CR46] Park CB, Cho N–H (1995). Consequences of son preference in a low-fertility society: imbalance of the sex ratio at birth in Korea. Population and Development Review.

[CR47] Pollard MS, Morgan SP (2002). Emerging parental gender indifference? Sex composition of children and the third birth. American Sociological Review.

[CR48] Poston DL (2002). Son preference and fertility in China. Journal of Biosocial Science.

[CR49] Rahman M, DaVanzo J (1993). Gender preference and birth spacing in Matlab, Bangladesh. Demography.

[CR50] Ray JG, Henry DA, Urquia ML (2012). Sex ratios among Canadian Liveborn infants of mothers from different countries. Canadian Medical Association Journal.

[CR51] Sen A (1990). More than 100 million women are missing.

[CR52] Seth S (2010). Skewed sex ratio at birth in India. Journal of Biosocial Science.

[CR53] Shu X (2004). Education and gender egalitarianism: the case of China. Sociology of Education.

[CR54] Singh N, Pripp AH, Brekk ET, Stray-Pedersen B (2010). Different sex ratios of children born to Indian and Pakistani immigrants in Norway. BMC Pregnancy and Childbirth.

[CR55] Statistics Sweden. (2003). *Access to microdata in the Nordic countries*. Stockholm: Statistics Sweden.

[CR56] Statistics Sweden (2009). Having children or not: Results from a questionnaire survey about women’s and men’s attitudes towards having children.

[CR57] Statistics Sweden. (2011). *Multi-generation register 2010: a description of contents and quality. background facts on population and welfare statistics, 2011, 2*. Stockholm: Statistics Sweden.

[CR58] Statistics Sweden. (2016a). *From Findland to Afghanistan—immigration and emigration since 1970 for persons born in different countries*. Örebro: Statistics Swden.

[CR59] Statistics Sweden 2016b. Immigrants aged 16–74 by gender, national background, educational level, education field, and country of origin.

[CR60] Statistics Sweden (2018). The future population of Sweden 2018–2070. Demographic reports.

[CR61] Teitelbaum MS, Mantel N (1971). Socio-economic factors and the sex ratio at birth. Journal of Biosocial Science.

[CR62] Terrell, M. L., Hartnett, K. P., & Marcus, M. (2011, 2011). Can environmental or occupational hazards alter the sex ratio at birth? A systematic review. *Emerging Health Threats Journal., 4*. 10.3402/ehtj.v4i0.7109. 10.3402/ehtj.v4i0.7109 PMC3168220 24149027

[CR63] Tonnessen M, Aalandslid V, Skjerpen T (2013). Changing trend? Sex ratios of children born to Indian immigrants in Norway revisited. BMC Pregnancy and Childbirth.

[CR64] UNFPA (2012). Sex imbalances at birth: current trends, consequences and policy implications.

[CR65] Van Larebeke NA, Sasco AJ, Brophy JT, Keith MM, Gilbertson M, Watersson A (2008). Sex ratio changes as sentinel health events of endocrine disruption. International Journal of Occupational and Environmental Health.

[CR66] Verropoulou G, Tsimbos C (2010). Differentials in sex ratio at birth among natives and immigrants in Greece: an analysis employing Nationwide micro-data. Journal of Biosocial Science.

[CR67] Von Borczyskowski A, Hjern A, Lindblad R, Vinnerljung B (2006). Suicidal behaviour in national and international adult adoptees: a Swedish cohort study. Social Psychiatry Psychiatric Epidemiology.

[CR68] World Health Organization (2011). Preventing gender-biased sex selection: an interagency statement. OHCHR, UNFPA, UNICEF, UN Women and WHO.

[CR69] Zeng Y, Tu P, Gu B, Xu Y, Li B, Li Y (1993). Causes and implications of the recent increase in the reported sex ratio at birth in China. Population and Development Review.

